# Oxford Nanopore enhanced accuracy of long-read amplicons applied to microbial whole-genome sequencing

**DOI:** 10.1128/spectrum.02856-25

**Published:** 2026-03-10

**Authors:** Marion Helsmoortel, Erwin Sentausa, Adrien Villain, Viet-Dung Tran, Emmanuelle Santiago-Allexant, Corinne Beaulieu, Amy Hesketh, Josephine Abi-Ghanem, Philippe Leissner, Adrien Saliou

**Affiliations:** 1 BIOASTER, Microbiology Technology Institute, Lyon, France; 2 bioMérieux SA, Marcy L’Etoile, France; 3 Lesaffre Institute of Science and Technology, Marcq-en-Barœul, France; Shandong University, Qingdao, China

**Keywords:** whole-genome sequencing, bloodstream infection (BSI), antimicrobial resistance (AMR), pathogen identification, blood culture, nanopore sequencing

## Abstract

The development of long-read sequencing technologies has enabled the analysis of extended nucleic acid sequences. These methods have proven their strength through their capacity to generate long reads, facilitating the analysis of complex genomic regions and rearrangements. Oxford Nanopore Technologies (ONT) offers a rapid and portable system that brings sequencing to the field. Although this is a great advantage for clinical settings, applications of long-read sequencing in this context have been limited by the high error rates reported for these methods. Here, we report an adaptation of an amplicon sequencing approach combined with unique molecular identifiers. We applied this method to whole-genome sequencing using mock community samples and human blood cultures spiked with common bloodstream infection pathogens. Our results showed a total error rate of <0.1% with V9 chemistry, which was further reduced by <0.05% when using the V14 chemistry. Our results also highlight the improvements of the V14 chemistry on the standard ONT ligation protocol and the importance of the basecalling tool for sequencing accuracy.

**IMPORTANCE:**

Recent advances in genome sequencing have greatly improved our ability to study microbes and detect infections. One such technology, Oxford Nanopore Technologies (ONT), can read long stretches of nucleic acids. ONT is also portable and can sequence in real time, making it useful in clinical settings. However, ONT accuracy is known to be lower than traditional short-read methods, limiting its widespread use. Fortunately, many strategies have emerged to overcome this limitation: better ONT chemistry, better basecaller, and hybrid approaches combining ONT with highly accurate short reads. Another promising method uses molecular barcodes or “Unique Molecular Identifiers” (UMIs) to make long reads at high accuracy, reaching accuracy levels similar to the existing short-read technologies. In our study, we optimized this UMI-based method and successfully applied it to human blood samples spiked with common infection-causing bacteria. The results showed a significant drop in ONT error rate, suggesting that this approach could make ONT a reliable tool for diagnosing infections and analyzing microbial DNA in clinical samples.

## INTRODUCTION

Long-read sequencing technologies have transformed genomics by enabling the analysis of extended nucleic acid sequences. Among these methods, Oxford Nanopore Technologies (ONT) offers the possibility to directly sequence native DNA and RNA long molecules. ONT overcomes short-read limitations by enabling the sequencing of repetitive regions along with their adjacent sequences within single, continuous reads. This facilitates the comprehensive detection of large-scale structural variations such as duplications and inversions, enhances the identification of horizontal gene transfer events, and enables the assembly of more complete and contiguous metagenome-assembled genomes and plasmids. At the taxonomic level, this improves the identification accuracy to species and strain levels. ONT also enables detailed analysis of transcript isoforms, including RNA modifications and poly(A) tails. Its real-time sequencing, portability, and adaptive sampling capabilities support on-site sequencing and efficient data collection, making ONT a valuable tool for microbial community analysis, pathogen detection, and genetic variation identification (1, 2).

Shotgun metagenomic sequencing (SMS) with short reads has been widely applied in clinical settings for infectious disease diagnosis, pathogen monitoring, and drug resistance profiling (3–6). Long-read sequencing, particularly ONT, has recently been explored for similar clinical applications (7). For example, it has been used for infectious disease diagnosis from respiratory samples (8), for detecting antimicrobial-resistance genes in stool (9), and for rapid sequencing of positive blood cultures to identify pathogens and assess antimicrobial resistance (AMR) (10–12). Despite these advances, ONT adoption in clinical diagnostics has been constrained by historically higher sequencing error rates compared with short-read platforms. Early assessments reported error rates as high as 40.2% (13), though recent improvements have reduced this to 10% or less (14, 15).

Several strategies have been developed by ONT to successfully improve sequencing accuracy. An early now-discontinued 2D library preparation protocol improved average accuracy from 86% to 94% by sequencing both strands of DNA molecules (16, 17). Accuracy further improved through iterative advances in flow cell design and sequencing chemistry: the R9.4.1 flow cell with kit V9 (SQK-LSK109) reached >97% accuracy, equivalent to a Phred score of Q16, while R10.4 combined with kit V12 (SQK-LSK112) achieved >99% (Q20) and enabled duplex sequencing, yielding Q30-level (>99.9%) consensus reads (18, 19). The latest R10.4.1/kit V14 combination achieves >99.5% accuracy (Q23) in simplex (20), though it still falls short of the near-perfect modal accuracy of the short-read Illumina sequencing (18).

ONT basecalling accuracy has also improved significantly. Guppy replaced Bonito as the default basecaller, introducing GPU acceleration and three accuracy-speed trade-off modes: fast, high accuracy (HAC), and super accuracy (SUP). Dorado, now the official ONT basecaller, further enhances speed and performance using newer neural network architectures (1, 21). In addition to basecalling improvements, numerous bioinformatics correction tools have been developed to further reduce ONT sequencing errors. Consensus-based tools such as Canu (22), Racon (23), and Medaka (24), as well as hybrid polishing methods like Pilon (25) and Nanopolish (26), can substantially improve consensus accuracy by correcting residual errors post-sequencing. Together, these computational and molecular approaches highlight that enhancing ONT accuracy relies on a combination of wet-lab and *in silico* strategies, which is particularly relevant for clinical metagenomics.

Despite these advancements, some challenges remain. For instance, PacBio—another long-read platform—still outperforms ONT with V14 chemistry and R10.4.1 flow cells in generating complete metagenome-assembled genomes and achieving higher accuracy, particularly in human gut microbiome studies (27). To further improve ONT sequencing, other methods have been proposed, such as hybrid sequencing. This approach takes advantage of the high accuracy of short-read sequencing to correct errors occurring in long-read sequencing. Accuracy can be increased from 81% to >99.9% but is not easily accessible due to the cost of generating sequence data from two sources (28).

**Fig 1:**
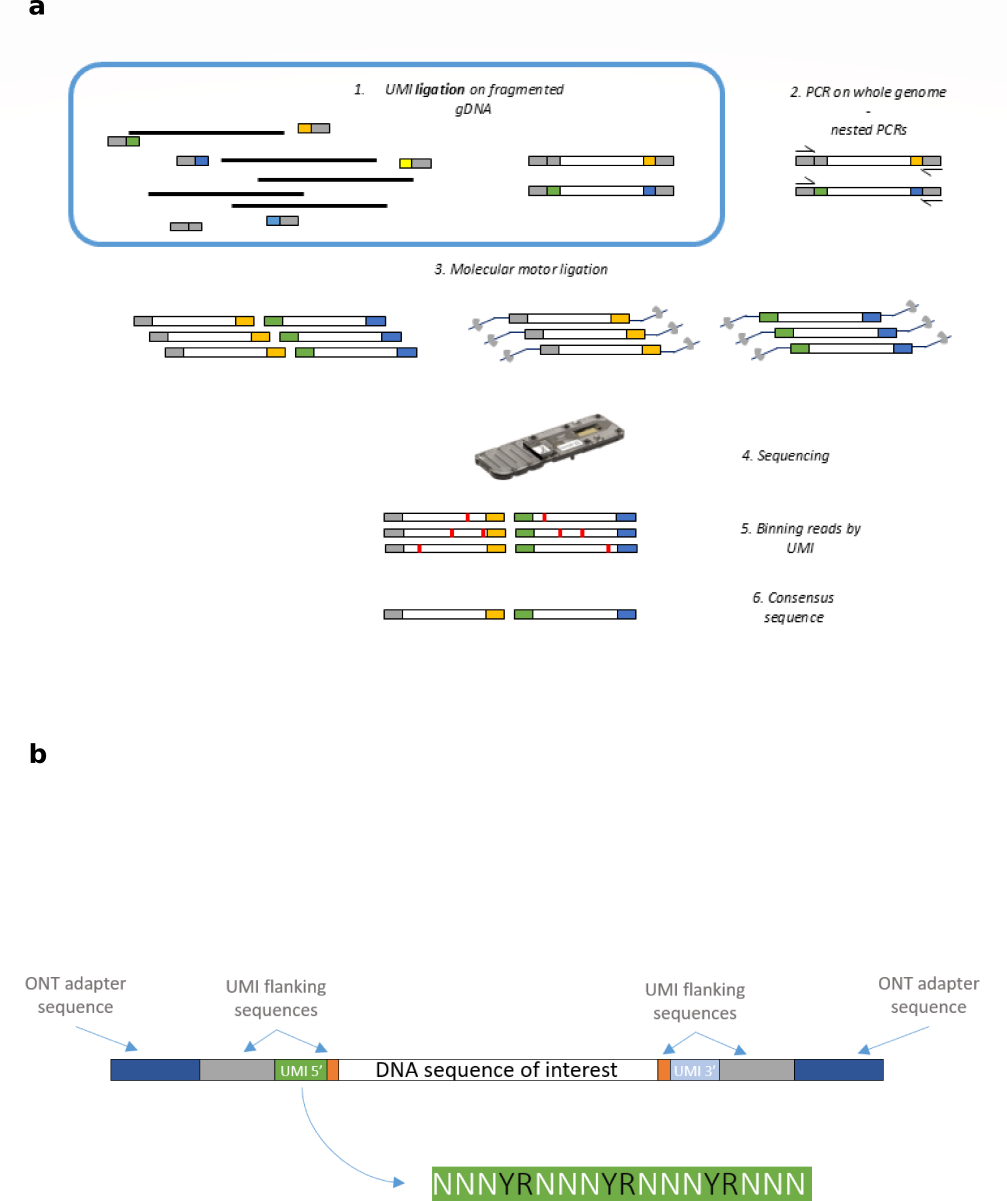
(**a**) Workflow for library preparation enabling error correction in whole-genome sequencing using the Oxford Nanopore Technologies (ONT) platform. First, genomic DNA (gDNA) fragments are ligated to adapters containing Unique Molecular Identifiers (UMIs). Second, PCR amplification enriches each UMI-tagged molecule, generating redundant copies. Third, ONT sequencing adapters containing molecular motors are ligated to these PCR amplicons. Fourth, the prepared library is loaded into a flow cell for sequencing. The final two steps involve bioinformatic analyses: reads are grouped (binned) based on shared UMIs, forming clusters representing the same genomic fragment with the typical ONT error rate. These clusters are subsequently processed to generate a consensus sequence for each UMI cluster, reducing errors and accurately representing the original genomic sequence. (**b**) Structure of reads after library preparation. Each DNA fragment is flanked symmetrically by similar adapter sequences. The read begins with the ONT adapter sequence, followed by a UMI-flanking sequence. The UMI consists of four repeats of random nucleotides, each interspersed with alternating purine and pyrimidine anchor nucleotides. A second UMI-flanking sequence immediately precedes the DNA fragment of interest.

Another developed strategy includes the use of unique molecular identifiers (UMIs), as demonstrated by Karst et al. (29). UMIs are short random sequences ligated to both ends of DNA fragments to uniquely tag each original molecule, enabling reads derived from the same template to be grouped and collapsed into a single consensus sequence (Fig. 1). This approach was initially developed to generate synthetic long reads using short-read sequencing (30). Karst et al. (29) combined UMIs with long-read sequencing to produce highly accurate consensus sequences. The authors generated over 25,000 UMI consensus sequences with a mean residual error rate of 0.0041%, corresponding to Q43. By enhancing ONT accuracy without requiring complementary short-read data, this method offers a more practical and cost-efficient solution for clinical workflows where rapid and stand-alone long-read analysis is desirable. Consequently, the UMI approach raises the ONT technology to a quality level comparable to short-read sequencing (29). More recent studies have applied the UMI-based approaches in various contexts, such as bacterial 16S amplicon sequencing (31, 32), human immunoglobulin heavy chain amplicon sequencing (31), antibody repertoire screening (33), variant detection and repeat quantification in cardiovascular risk marker (34), and sperm mosaicism detection (35). However, these methods have not been applied to bacterial whole-genome sequencing (WGS) or SMS.

In this study, we optimized the UMI-based protocol developed by Karst et al. (29) and successfully applied it to human blood cultures spiked with common bloodstream infection pathogens. Our optimized protocol markedly reduced ONT’s error rate, demonstrating its potential to bridge the gap between technological advancements and practical clinical utility, particularly in clinical SMS applications.

## MATERIALS AND METHODS

### Sequence design and oligo synthesis

The short adapter and corresponding primer sequences used were identical to those described by Karst et al. (29) and are detailed in Table S1. Longer adapter sequences and their corresponding primers were newly designed based on the *HUN1* gene of *Arabidopsis thaliana* to ensure sufficient phylogenetic distance from bacterial genomes. These sequences are also listed in Table S1. All oligonucleotides were synthesized by Integrated DNA Technologies using standard desalting purification.

### DNA material

Developments were conducted with ZymoBIOMICS HMW DNA standard (D6322, Zymo Research) containing eight microbial species (including yeast). Species composition and theoretical abundance ratios are summarized in Table S2. The optimized protocol was validated using genomic DNA from bacterial-spiked blood cultures. Two independent experiments were conducted, each testing six strains from clinically relevant bloodstream pathogens. The first experiment (using ONT V9 chemistry) included two strains of each of the following bacteria: *Staphylococcus aureus*, *Escherichia coli*, and *Klebsiella pneumoniae*. In the second experiment (using updated ONT V14 chemistry), one *K. pneumoniae* strain was substituted with *Pseudomonas aeruginosa*, and an alternative *S. aureus* strain was included.

### Spiking of blood cultures and DNA extraction

A fresh bacterial suspension was prepared to spike 50 µL in a blood tube (EFS EDTA 2K tube from healthy donor), equivalent to 100 CFU. The FA Plus (410851, bioMérieux) blood culture bottle is spiked with the full blood tube. Blood culture bottles were incubated in the BACT/ALERT system (bioMérieux) until positivity was detected by the system. Once sample positivity was reached, DNA was extracted by bioMérieux as described by Azami et al. (36). Briefly, a mechanical lysis was applied on positive blood culture before extraction with ZymoBIOMICS DNA miniprep kit (D4300, Zymo Research).

### Sample preparation: DNA fragmentation

Mechanical fragmentation involved hydrodynamic shearing in Covaris g-TUBEs (520079, Covaris) using benchtop centrifugation. In the development and validation of the protocol, 150 ng of DNA was diluted to a final volume of 50 µL with DNase/RNase-free water and loaded into a Covaris g-TUBE. A centrifugation speed of 7,500 rpm for 1 minute was applied to obtain 8 kb fragments. A size-selection step was introduced immediately after DNA fragmentation to remove fragments smaller than 4 kb. This step involved PEG precipitation using the Short Read Eliminator XS kit (102-208-200, PacBio). Briefly, 60 µL of Buffer SRE XS was mixed with 60 µL of DNA (25–150 ng/µL) and centrifuged at 10,000 × *g* for 30 minutes at room temperature. After carefully discarding the supernatant, the pellet was washed twice with 200 µL of 80% ethanol (centrifugation at 10,000 × *g*, 2 minutes), air-dried for 1 minute, and resuspended in 30 µL elution buffer preheated to 50°C. Complete resuspension of DNA was achieved after a 20-minute incubation at room temperature for an average yield of 100 ng at the end of the procedure.

### Quantifications and size quality controls

DNA concentrations were measured using the Qubit dsDNA HS Assay (Q32851, Thermo Fisher Scientific) on a Qubit 2.0 fluorometer (Thermo Fisher Scientific). Fragment sizes were assessed using the HS Large Fragment 50 kb kit (DNF-464-0500, Agilent) on a Fragment Analyzer 5200 (Agilent).

### DNA purification and size selection

Sample purification was performed using Clean NA magnetic beads (CNGS-0050, Proteigene). The bead-sample mixture was incubated for 15 minutes at 25°C in a thermal cycler with the lid open. The tubes were briefly centrifuged, placed on a magnetic rack for 2 minutes, and the supernatant was discarded. Beads were washed twice with 180 µL of 80% ethanol, then resuspended in the desired volume of elution water. To enhance recovery of larger fragments, beads were incubated at 37°C for 5 minutes for elution. The tubes were briefly centrifuged and placed on a magnetic rack for 2 minutes, and the elution volume was recovered.

### UMI tagging of fragmented DNA

Before ligation of UMI adapters, an end-prep was performed with UltraII End-prep buffer and 3 µL of UltraII End-prep enzyme (E7546, New England Biolabs) according to the manufacturer’s instructions with 100 ng of DNA as input. The repaired DNA was purified with magnetic beads at a 1:1 ratio.

Adapters were delivered as single-stranded DNA and required hybridization before ligation to genomic DNA. The hybridization mixture consisted of 5 µL of adapter containing UMIs at 100 µM, 5 µL of short adapter at 100 µM, 25 µL of T4 DNA ligase buffer, and DNase/RNase-free water to achieve a final volume of 250 µL. This mixture was heated to 95°C for 5 minutes in a thermoblock, after which the thermoblock was switched off and the mixture was allowed to gradually cool overnight.

A ligation reaction was performed by combining 15 µL of end-repaired DNA with 10 µL of the hybridized adapter mix and 25 µL of Blunt/TA Ligase Master Mix (M0367, New England Biolabs). After 30 minutes of incubation at 25°C in a thermal cycler with an open lid, the products were purified using magnetic beads at a 1:1 ratio.

### Library amplification

Three different DNA polymerases were extensively evaluated for PCR amplification: Platinum SuperFi II (12369010, Thermo Fisher Scientific), Kapa HiFi Hot Start DNA polymerase (07958897001, Roche Diagnostics), and LongAmp Taq DNA polymerase (M0323, New England Biolabs).

A range of DNA inputs was tested, from 0.1 to 100 pg added in the first PCR. Each experiment included controls (data not shown): a negative control and a 1 ng input control, expected to be inefficient for UMI redundancy due to excessive diversity in the number of UMIs.

For Platinum SuperFi II, PCR reactions were conducted using 1 µM of primer lu_pcr_fw_v7 or specifically adapted primers with the longer adapters (see Table S1) combined with 1× green PCR master mix. Reaction volumes were 15 µL for the initial amplification and increased to 50 µL for subsequent amplifications (PCR2 and PCR3). Cycling involved an initial denaturation at 98°C for 20 seconds, followed by a two-stage amplification protocol: the first stage consisted of five cycles at 98°C for 20 seconds, 60°C for 15 seconds, and 72°C for 8 minutes; the second stage included five cycles at 98°C for 20 seconds and 72°C for 8 minutes, with a final extension at 72°C for 6 minutes. The three PCR methods differed by their number of cycles.

Reactions using Kapa HiFi Hot Start DNA polymerase had a total volume of 100 µL, split equally into two tubes. The reaction mix comprised 1× KAPA HiFi buffer, 0.3 mM dNTPs, 0.6 mM forward primer, 1 mM MgCl_2_, and 0.5 U polymerase. Cycling started with an initial denaturation at 95°C for 3 minutes, followed by 35 cycles at 98°C for 20 seconds, 60°C for 20 seconds, and 72°C for 5, 6, or 8 minutes, concluding with a final extension at 72°C for 5, 6, or 8 minutes. Successive amplifications included similar cycle patterns (10, 30, or 8 cycles) after the initial denaturation step, always concluding with a final extension.

PCR amplifications using LongAmp Taq DNA polymerase were carried out initially in a total volume of 50 µL, with subsequent amplifications performed in two separate 50 µL reactions. The reaction composition included 1× LongAmp reaction buffer, 0.8 µM primers, 300 µM dNTPs, and 0.1 unit/µL polymerase. The cycling conditions consisted of an initial denaturation at 94°C for 30 seconds, followed by 10, 30, or 8 cycles of denaturation at 94°C for 15 seconds, annealing at 60°C for 15 seconds, and elongation at 65°C for 5 minutes, ending with a final extension at 65°C for 6 minutes. PCR products were purified after each amplification using magnetic beads at a ratio of 0.75×.

### Library preparation and sequencing

Amplicons were prepared for sequencing using the Ligation Sequencing Kit V9 (SQK-LSK109, Oxford Nanopore Technologies) combined with barcoding extension. Barcodes were selected from kits available in batches of 12 (EXP-NBD104 or EXP-NBD114, Oxford Nanopore Technologies). The library preparation protocol was performed with 150 ng of DNA as input when multiplexing several samples and up to 1 µg for single-sample runs. The protocol followed was “Native barcoding genomic DNA” (versionNBE_9065_v109_revAG_14Aug2019), with minor modifications. Briefly, AMPure XP beads were substituted with Clean NA beads (CNGS-0050, Proteigene) during all purification steps. Binding steps were consistently carried out at 25°C for 15 minutes in a thermal cycler with an open lid. For elution, tubes were incubated at 37°C for 5 minutes in a thermal cycler with the lid open. The final library pool was quantified and assessed for quality via capillary electrophoresis. For sequencing, 50 fmol of the prepared library were loaded onto an R9.4.1 flow cell on a GridION X5 (Oxford Nanopore Technologies Ltd), employing high-accuracy basecalling using Guppy 3.2.1, 4.0.11, 4.2.3, 4.3.4, 5.0.11, and 5.0.13.

For the experiment with the V14 chemistry of ONT, the Native Barcoding Kit 96 V14 (SQK-NBD114.96, ONT) was used with the latest version of the protocol (NBE_9171_v114_revR_30Jan2025). The same modifications as in the V9 protocol were applied. The final library pool was quantified and assessed for quality via capillary electrophoresis. For sequencing, 50 fmol of the prepared library was loaded onto an R10.4 flow cell on a GridION mk1 (Oxford Nanopore Technologies Ltd). Live basecalling was performed using Dorado Basecall Server 7.6.7 using high-accuracy setting, and the same data were later rebasecalled using super-accuracy setting.

### Bioinformatics analysis

The longread_umi nanopore_pipeline v0.3.2 (https://github.com/SorenKarst/longread_umi [29]) was used for trimming UMIs, binning reads into UMI clusters, and subsequently building consensus sequences (polished with Racon [23]). Log files were parsed to extract quality-control metrics, including read trimming statistics, UMI cluster counts and size distribution (<10, 10–50, >50 reads), per-sample yield (% reads retained, UMIs, or consensuses per 1,000 reads), and consensus properties (number, mean length, coverage, and Qualimap error rate). Additional flags identified UMI extraction, read orientation, or barcode check failures. No strict thresholds were applied, but runs with <50% read retention, <30% of UMIs in the 10–50 range, or error rates >0.3% at <3× coverage were considered suboptimal.

Raw sequencing data obtained from ONT Ligation data sets and consensus sequences generated from UMI data sets were mapped to reference genomes, specifically the ZymoBIOMICS HMW DNA Standard and Illumina draft assemblies for spiked blood cultures. Mapping was performed using minimap2 v2.17-r941 (37) with the preset -x map-ont, using alignment output format (-a) and long CIGARs (-L). Consensus sequences were mapped with the same parameters plus the --cs=short option. Resulting BAM files were sorted and indexed using samtools (38). Mismatch, insertion, deletion, and total error rates were estimated from mapping results using Alfred v0.2.8 (39) (qc mode) with the corresponding reference genomes, and depth of coverage was assessed using Qualimap v2.2.2-dev (40) (bamqc, default parameters).

## RESULTS

### Optimized UMI-based protocol workflow for accurate consensus generation

The crucial step for adapting the UMI-based protocol to whole-genome sequencing is the library preparation step. The corrective library preparation method relies on ligating synthetic DNA fragments carrying UMIs. Post-sequencing, reads are grouped based on pairs of UMIs, with each cluster representing an original genomic DNA molecule. If a cluster reaches a sufficient number of sequences, a high-quality consensus sequence is generated, accurately reflecting the original molecule’s sequence. Through this study, we optimized the UMI-based protocol and determined the optimal conditions for consensus sequence generation for accurate pathogen identification. Optimizations involved evaluating factors critical for amplifying enough DNA fragments tagged with UMIs and enriching each UMI effectively. Adapter sequence and length, PCR enzyme, and initial DNA quantities were identified as essential parameters. The developed protocol workflow is illustrated in Fig. 1a. The sequenced reads comprise, from 5′ to 3′: the ONT adapter, a first flanking sequence which is a generic primer binding region for subsequent PCR amplifications, the UMI sequence, another flanking sequence, the genomic sequence, and finally, a second adapter in the opposite order with a different UMI (Fig. 1b). After linking these adapters to randomly fragmented genomic DNA, multiple PCR amplification steps are performed to enrich each DNA molecule, which becomes labeled with distinct UMIs at both the 5′ and 3′ ends. Subsequently, sequencing adapters equipped with molecular motors provided by ONT are ligated. The resulting library is then sequenced in ONT flow cells on either a MinION or GridION.

### Longer adapters reduce recovery of UMI-containing reads

**Fig 2:**
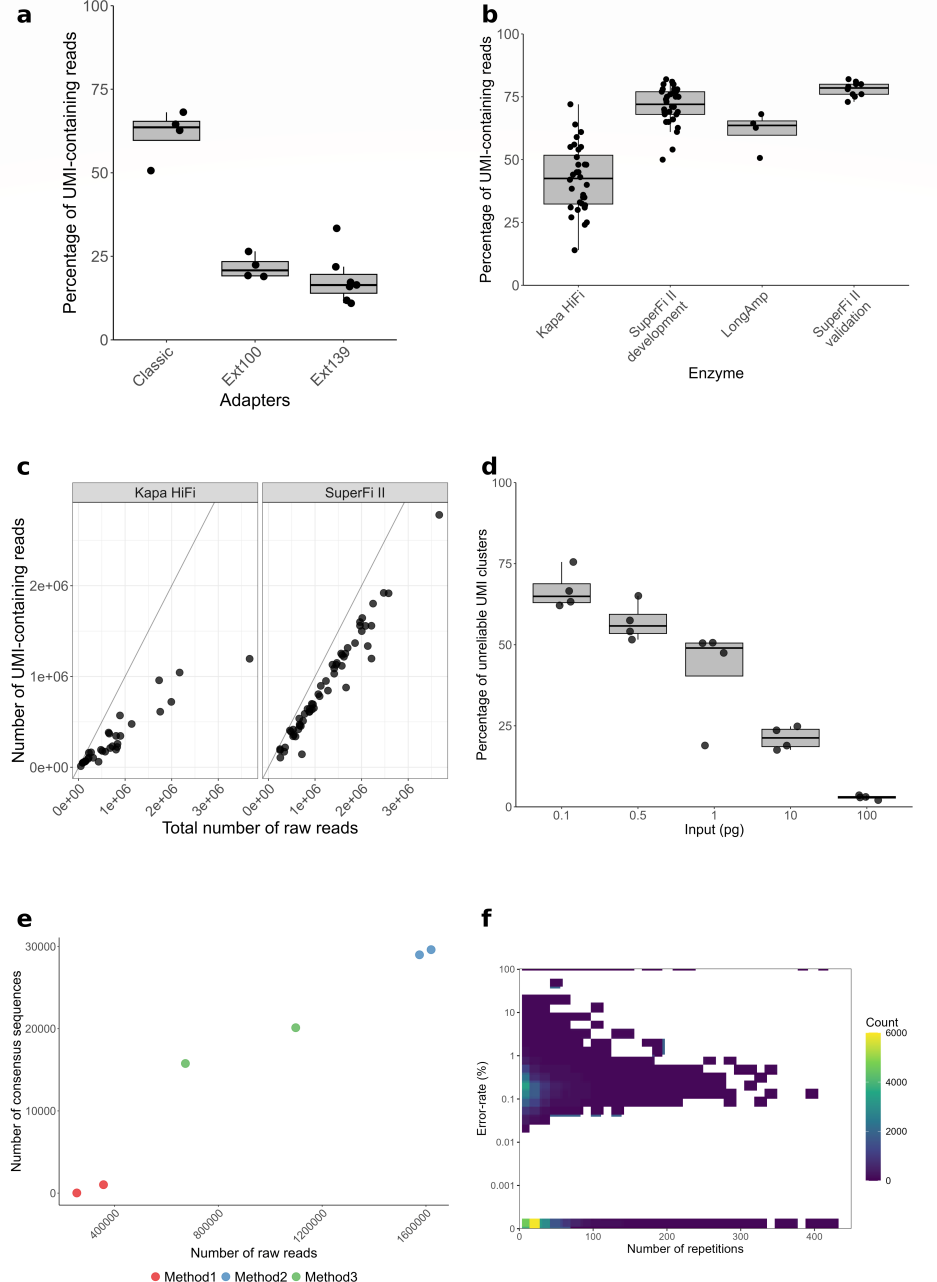
Comparison of different experimental conditions for optimal UMI amplification and cluster generation. (**a**) Percentage of sequenced reads carrying UMIs for different adapter lengths: classic (61 bp) and adapters carrying extensions of 100 bp (Ext100) or 139 bp (Ext139). (**b**) Percentage of reads carrying UMIs using different polymerases for enrichment steps: Kapa HiFi polymerase (*n* = 32), SuperFi II (*n* = 32), and LongAmp polymerase (*n* = 4). The final validation experiment using SuperFi II polymerase is shown separately. (**c**) Scatter plot for correlation between the number of total raw reads and UMI-containing reads using Kapa HiFi or SuperFi II. The line represents a perfect correlation (*R*^2^ = 1) between the two values and is the target of optimizations. (**d**) Impact of the initial DNA input (0.1–100 pg) on the percentage of UMI clusters which could not generate a consensus sequence, qualified as unreliable clusters, versus the total number of clusters generated. These unreliable clusters are characterized by too few or too many sequences. (**e**) Comparison of consensus sequences generated versus total raw reads using three different PCR methods of amplification as detailed in the Materials and Methods section. (**f**) Two-dimensional density heatmap illustrating the relationship between UMI cluster size (number of repetitions) and error rate. The color scale represents the frequency of observations for each bin. The *y*-axis is on a log scale.

The protocol was developed for 18 nucleotides-long UMIs with interspersed anchor and random nucleotides. As illustrated in Fig. 1a, a generic sequence of 24 bp is added upstream (5′) of each UMI to allow primer binding during PCR amplifications. In final libraries, with the ONT adapter, the UMI is contained in the first hundred base pairs of each read. However, previous internal experiments (data not shown) or studies (14) showed higher error rates at the 5′ end of ONT reads with *Q* scores varying a lot between Q5 and Q25 within the first 100–150 nucleotides. To address this, two extended adapter versions were designed, adding either 100 bp (Ext100) or 139 bp (Ext139) to position the UMI in a region of the reads where the Q score is more stable. The performance of the extended adapter was compared to the original shorter adapter using different PCR enzymes. As shown in Fig. 2a, both Ext100 and Ext139 reduced the percentage of reads containing UMIs compared to the shorter adapter. Therefore, the original shorter adapter was retained for subsequent experiments.

### SuperFi II consistently generates high percentages of UMI-containing reads

The highest proportion of reads containing UMIs is critical for successful consensus sequence generation. Polymerases used in enrichment PCR notably affect the ratio of raw reads to UMI-containing reads. Three polymerases—Kapa HiFi, LongAmp, and SuperFi II—were compared based on UMI recovery efficiency (Fig. 2b). SuperFi II polymerase showed the highest performance, with 71.4% ± 7.5% of reads containing UMIs (*n* = 32), surpassing Kapa HiFi, which generated 42.3% ± 13.1% (*n* = 32). SuperFi II results were further validated in a second independent experiment (Fig. 2c, SuperFi II validation sample). LongAmp demonstrated promising results in a limited set of experiments, with 61.5% ± 7.5% UMI-containing reads across four samples only. However, early observations revealed high variability and poor reproducibility in the initial assays. Based on these findings and considering limitations in available DNA material and experimental time, we decided not to pursue further testing with LongAmp. Figure 2c illustrates the relationship between the number of total reads and reads containing UMIs, highlighting that SuperFi II achieved a higher proportional increase in UMI-containing reads compared to Kapa HiFi. An outlier sample was detected, and results would need to be confirmed for the LongAmp experiment for which data are not shown.

### UMI cluster generation is improved with increased DNA input

The number of DNA molecules used in PCR amplification impacts both the frequency of repeated fragments and rare events (single-read occurrences), as well as the cluster size for each UMI-tagged DNA molecule (UMI cluster). The objective of varying DNA input is to define conditions in which good quality clusters are obtained, with enough repeated reads (>10) and as few as possible of unreliable clusters. These unreliable clusters are defined as clusters containing only one read or, on the contrary, too many for consensus sequence generation and analysis (>100,000). DNA inputs ranging from 0.1 to 100 pg were tested, which is equivalent to approximately 2.3 *×* 10^4^ to 2.3 *×* 10^7^ DNA molecules. The optimal input quantity for generating robust consensus sequences was defined by measuring the number of good quality clusters versus unreliable clusters. We demonstrated that 100 pg DNA input yielded the lowest rate of unreliable clusters (<10%), with this rate increasing proportionally as the DNA input decreased (Fig. 2d), along with a reduction in total valid clusters (data not shown). A classic range of consensus obtained was between 10,000 and 30,000 consensus sequences, varying with PCR conditions or polymerases and sequencing depth.

### Adjusting PCR conditions increases the number of UMI clusters

The effect of varying PCR conditions on the yield of consensus sequences was also evaluated. Figure 2e shows that the highest number of consensus sequences was achieved with a protocol comprising three consecutive PCRs of 10, 30, and 8 cycles (method 2), which also produced the highest overall yield. Indeed, 1,597,441 ± 31,638 raw reads were obtained, leading to the generation of 29,299 ± 3,078 consensus sequences. Two alternative PCR methods, one using two PCR steps of 10 and 30 cycles (method 1) and another with 35 and 8 cycles (method 3), resulted in 306,082 ± 72,699 and 966,279 ± 19,771 raw reads, respectively. From these, 524 ± 709 and 17,942 ± 3,078 consensus sequences were generated.

### Consensus generation significantly corrects the ONT error rate with V9 and V14 chemistry

**Fig 3:**
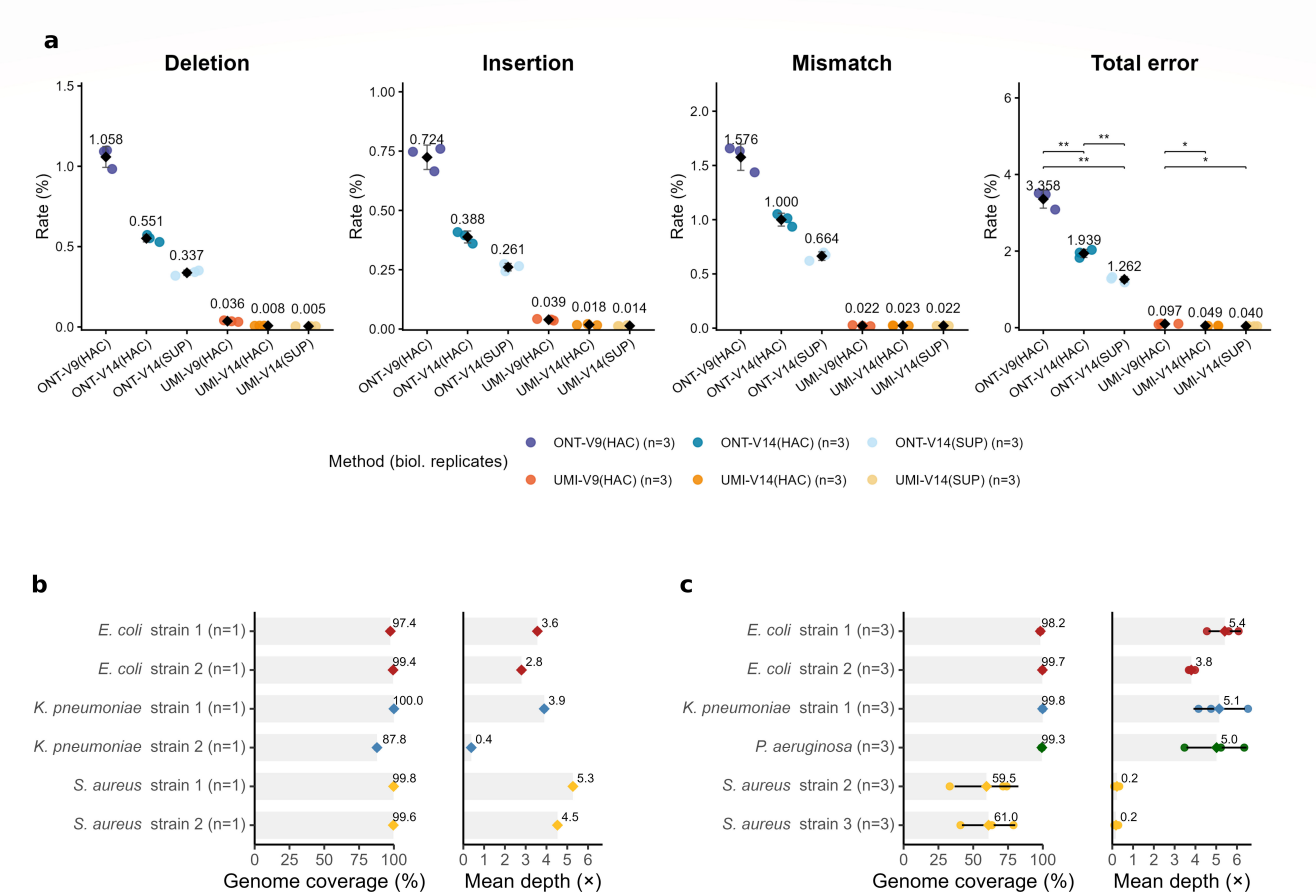
(**a**) Error rates of ONT sequencing and UMI consensus sequences with V9 and V14 chemistries. Deletion, insertion, mismatch, and total error rates are shown for ONT ligation-based sequencing and UMI consensus sequences obtained using high-accuracy (HAC) and super-accuracy (SUP) basecalling modes. Each point represents an individual biological replicate (*n* = 3 per condition); black diamonds indicate mean values. Across all conditions, UMI consensus sequences consistently achieve substantially lower error rates than classic ONT sequencing. Statistical analysis was performed using pairwise *t*-tests (Holm corrected) for total error rates. Significance is indicated as follows: *P < 0.05, **P < 0.01. The difference between UMI V14 (HAC) and UMI V14 (SUP) was not significant (*P* = 0.055). (**b**) Validation of the sequencing and consensus protocol on artificially inoculated human blood cultures using the ONT V9 chemistry. Genome coverage (%) and mean sequencing depth (×) are shown for two distinct strains each of *Escherichia coli*, *Klebsiella pneumoniae*, and *Staphylococcus aureus*. Each colored diamond represents the single observed value per strain (*n* = 1). (**c**) Validation of the protocol on artificially inoculated human blood cultures using the updated ONT V14 chemistry. Genome coverage (%) and mean sequencing depth (×) are shown for *E. coli*, *K. pneumoniae*, *Pseudomonas aeruginosa*, and *S. aureus*. Dots represent technical replicates (*n* = 3); black horizontal lines show the standard deviation; and colored diamonds indicate the mean values for each strain. Due to limited DNA availability, one *K. pneumoniae* strain was replaced with *P. aeruginosa*, and a third distinct *S. aureus* strain (strain 3) replaced the original strain 1.

The size of UMI clusters required for optimal consensus generation was evaluated since the number of sequences per consensus directly influences sequencing duration and multiplexing capability. Most error-free sequences were generated from clusters containing fewer than 50 sequences (Fig. 2f). Among these UMI clusters of fewer than 50 sequences, the highest density was associated with a base error rate of approximately 0.1%. This finding was confirmed using samples from a microbial community standard with known reference genomes. In comparison, standard ONT whole-genome sequencing typically showed base mismatch, insertion, and deletion error rates exceeding 1% for each. The total error rate with the V9 chemistry of ONT sequencing was 3.36%. This rate was reduced to 1.9% by the technological improvements of the V14 chemistry. The SUP basecalling model using Dorado was compared to the HAC method using Guppy formerly used with V9 chemistry. The 1.9% error rate obtained with the V14 chemistry and the HAC basecaller was further reduced when using the SUP basecaller, bringing the error rate down to 1.26%. In contrast, the total error rate observed for samples processed with UMIs was substantially lower. With V9 chemistry, the total error rate was 0.097% and showed the same tendencies with the V14 chemistry with 0.049% and 0.040% with HAC and SUP basecallers, respectively (Fig. 3a).

### Performance evaluation using spiked human blood cultures in a clinical sequencing context

The protocol was validated in a clinically relevant context using human blood cultures artificially inoculated with three common bloodstream pathogens: *Escherichia coli*, *Klebsiella pneumoniae*, and *Staphylococcus aureus*. The method efficiently recovered near-complete genome coverage for two distinct strains of each species (Fig. 3b). Mean genome coverage exceeded 97% for most samples using consensus sequences, although one strain (*K. pneumoniae* strain 2) exhibited slightly lower coverage at 87.8%. Depth of coverage based on consensus sequences ranged typically between 2.8× and 5.3×, with the same *K. pneumoniae* strain showing notably lower coverage at 0.4×. The reduced coverage was due to insufficient sequencing depth for this sample on one flow cell; the cause could not be determined, and resequencing was not possible because no amplicons remained.

An additional experiment was conducted using ONT V14 chemistry to evaluate performance improvements associated with updated sequencing chemistry combined with UMI clustering. Due to limited DNA availability, one *K. pneumoniae* sample was substituted with *Pseudomonas aeruginosa*, and the first *S. aureus* strain was replaced with an alternative *S. aureus* strain. Results for *E. coli* and *K. pneumoniae* were consistent with expectations, achieving genome coverage exceeding 98% and consensus sequence depth ranging from 3.8× to 5.4×. The substituted *P. aeruginosa* sample exhibited high genome coverage at 99.3%, with a consensus depth of approximately 5×. However, performance for both *S. aureus* strains was unexpectedly poor, with consensus mapping covering only up to 61% of the genomes at a shallow depth of 0.2× (Fig. 3c). This reduction is likely attributed to prolonged storage, as these samples experienced an extended delay between DNA extraction and sequencing with V14 chemistry. We believe this delay compromised DNA quality, reducing the ligation efficiency of UMIs.

## DISCUSSION

As point-of-care diagnostics continue to gain importance for the rapid identification of bloodstream pathogens, ONT sequencing represents an attractive platform due to its portability and ease of use. However, its relatively high basecalling error rate remains a significant challenge, particularly when accurate species-level identification and detection of AMR genes are required (36).

In this study, we demonstrate that integrating UMIs into the ONT library preparation workflow significantly improves sequence accuracy by reducing error rate through consensus building. Our protocol was systematically optimized to ensure high efficiency of error correction with minimal sequencing throughput, thereby offering a cost-effective alternative to traditional long-read WGS approaches.

A key optimization involved the design and length of UMI-adapter constructs. We observed that when the total adapter length exceeded 61 bp, UMI recovery efficiency from raw reads declined markedly. This observation was critical for maximizing downstream UMI-based consensus calling. Furthermore, we evaluated three DNA polymerases for amplification efficiency and consistency and found that Platinum SuperFi II polymerase yielded the most robust and reproducible results in terms of UMI redundancy, a crucial parameter for accurate consensus sequence generation.

The amount of input DNA also proved to be a critical parameter in the success of the protocol. We determined that 100 pg of bacterial genomic DNA provided optimal results for whole-genome amplification in low-complexity samples (1–12 pure bacteria species per sample or one species with variable amounts of host DNA). These parameters, when incorporated into our workflow, contribute to achieving a robust and scalable method for UMI tagging and library preparation compatible with ONT sequencing.

Cluster generation from UMI-tagged reads remains a bottleneck, as the number of reads per UMI cluster is directly tied to consensus generation. Drawing on previous findings from Volden et al. (41), who reported that at least 10 reads per cluster were required for reliable consensus generation on PacBio platforms, we applied a similar threshold in our analysis pipeline. Our data support this criterion: most high-quality, error-free sequences originated from clusters composed of 10–50 reads. Notably, we found diminishing returns in accuracy beyond this range, suggesting an optimal trade-off between throughput and accuracy.

Nevertheless, generating multiple reads per UMI cluster inherently demands more sequencing resources than standard single-read ONT workflows, potentially increasing per-sample costs. Using our optimized protocol, over 80% of reads successfully retained UMI sequences, and more than 90% of resulting clusters produced high-confidence, error-free consensus sequences suitable for downstream species identification and resistance gene characterization. However, compared with conventional ONT or short-read workflows, amplicon preparation is longer and more labor intensive, requiring controlled fragmentation, UMI tagging, and multiple PCR amplification steps. This extended workflow increases hands-on time and requires more technical expertise to maintain reproducibility. In addition, consensus generation and UMI clustering require significant computational resources, which can extend the time to result by several hours compared with standard ONT pipelines.

Another limitation concerns the diversity and complexity of the samples that can be effectively processed. The method performs optimally for low- to moderately complex samples, where distinct UMI clusters can be accurately formed and analyzed. Despite these constraints, the approach provides a strong balance between accuracy and sensitivity, enabling robust sequence reconstruction from very low amounts of input DNA, an important advantage for degraded, low-yield, or clinically challenging specimens. Continuous improvements in ONT technologies (including flow cell design, sequencing chemistry, and basecall algorithms) are progressively enhancing the accuracy of nanopore long-read sequencing. The recent basecaller Dorado incorporates a “super-accurate” model to call bases, increasing raw read accuracy close to 99%. In this study, we observed similar results with an error rate of 1.2% with our reference samples. This baseline accuracy is advantageous for UMI consensus achieving a mean error rate of 0.04%, while consensus sequences obtained with V9 chemistry achieved 0.1%. New chemistries are still improving, but UMIs demonstrate advantages for the identification of chimeric reads and rearrangements with reliability since DNA is marked before any of the amplification steps (42, 43). As ONT technology continues to evolve, it is likely that the combination of improved base calling and robust UMI strategies will further close the gap between nanopore and short-read technologies for clinical-grade microbial genomics.

Recent studies have further highlighted the importance of improving sequencing accuracy and assembly strategies for microbial and viral characterization. For instance, Cook et al. (44) demonstrated that ONT coupled with Illumina sequencing can reveal distinct viral populations in human gut samples. Similarly, Chakrawarti et al. (45) showed that hybrid Illumina–ONT assemblies significantly enhance multilocus sequence typing and antimicrobial resistance gene detection in *S. aureus* isolates compared with Illumina-only or ONT-only assemblies. These findings reinforce the value of approaches that combine or correct long reads in complex or clinically relevant microbial analyses.

When applied to clinical samples, our approach shows that even at relatively low sequencing depth, the quality of the recovered consensus sequences is sufficient to support robust taxonomic identification. Importantly, the high fidelity of UMI-corrected sequences opens avenues for downstream applications such as antimicrobial susceptibility testing and AMR detection directly from blood-derived bacterial DNA. However, the success of this approach remains highly dependent on the quality and integrity of the input DNA, which can be a limiting factor in certain clinical contexts (host DNA contamination or degraded samples). In addition, the implementation of UMI-based workflows still requires specific equipment and technical expertise, which may represent a practical limitation for their application in clinical environments. To test those limitations, the application of the UMI method should be tested on a larger set of fresh clinical samples. Such evaluations will also help determine whether the increased hands-on and computational processing times of UMI workflows remain compatible with rapid time-to-result requirements in diagnostic settings.

Altogether, our findings support the integration of UMI-enhanced nanopore sequencing as a promising tool for accurate, real-time pathogen identification in infectious disease diagnostics.

## Data Availability

Raw nanopore sequencing reads have been deposited in the NCBI Sequence Read Archive under BioProject accession number PRJNA1285122 (BioSample accession numbers SAMN49750384–SAMN49750510 and SRA accession numbers SRR34331949–SRR34332075).

## SUPPLEMENTAL MATERIAL

Supplemental tables spectrum.02856-25-s0001.pdf Tables S1 and S2.

ASM does not own the copyrights to Supplemental Material that may be linked to, or accessed through, an article. The authors have granted ASM a non-exclusive, world-wide license to publish the Supplemental Material files. Please contact the corresponding author directly for reuse.
