# Supplementary material for: Oxford Nanopore enhanced accuracy of long-read amplicons applied to microbial whole-genome sequencing
Source: Microbiol Spectr. 2026 Mar 10;14(4):e02856-25. doi: 10.1128/spectrum.02856-25 (PMC13055378; doi:10.1128/spectrum.02856-25)
Supplement: Supplemental tables — Tables S1 and S2. [file spectrum.02856-25-s0001.pdf]

Supplemental materials

Table S1

Primer and adapter sequences; N corresponds to any nucleotide incorporated randomly; Y corresponds to C or T, R corresponds to A or G randomly incorporated as well. Five prime extremity of Lu_adp_s_v4 oligonucleotide is phosphorylated (p). An asterisk in the 3’ last Thymine of the UMI oligo indicates this base is modified by addition of a phosphorothioate group

Primer name	Sequence (5’-3’)
lu_adp_l_v4	CAAGCAGAAGACGGCATACGAGATNNNYRNNNYRNNNYRNNNACGTGTGCTCTTCCGATC*T
Lu_adp_s_v4	^{P}GATCGGAAGAGCACACGT
Lu_pcr_fw_v7	CAAGCAGAAGACGGCATACGAGAT
Adapter_Ext100	TGTTTAGATCAATCCGTCTCGAACCCAGGGGTTAGTATATAGTGGATCAAACTGATCCGACCCGA<br>CCCGACCCGAATTATTAATGTCGGAAGTTAATGAGCAAGCAGAAGACGGCATACGAGATNNNYRN<br>NNYRNNNYRNNNACGTGTGCTCTTCCGATC*T
Adapter_Ext139	CGATTCCACTCTCTTGATTGCTGTTTAGATCAATCCGTCTCGAACCCAGGGGTTAGTATATAGTGGA<br>TCAAACTGATCCGACCCGACCCGACCCGAATTATTAATGTCGGAAGTTAATGAGATGAGTGGTGGT<br>TCTATTCAAGCAGAAGACGGCATACGAGATNNNYRNNNYRNNNYRNNNACGTGTGCTCTTCCGATC*T
Primer1_Ext100	TGTTTAGATCAATCCGTCTCG
Primer2_Ext100	AGATCAATCCGTCTCGAACC
Primer3_Ext100	CAATCCGTCTCGAACCCAGG
Primer1_Ext139	CGATTCCACTCTCTTGATTGC
Primer2_Ext139	TTCCACTCTCTTGATTGCTGT
Primer3_Ext139	TCCACTCTCTTGATTGCTGTTTAG

Table S2

Theoretical genomic DNA composition of seven bacteria and one yeast in ZymoBIOMICS® HMW DNA Standard

Species	Theoretical genomic DNA composition (%)
Pseudomonas aeruginosa	14
Escherichia coli	14
Salmonella enterica	14
Enterococcus faecalis	14
Staphylococcus aureus	14
Listeria monocytogenes	14
Bacillus subtilis	14
Saccharomyces cerevisiae	2
